# Hughes Abdominal Repair Trial (HART) – Abdominal wall closure techniques to reduce the incidence of incisional hernias: study protocol for a randomised controlled trial

**DOI:** 10.1186/s13063-016-1573-0

**Published:** 2016-09-15

**Authors:** J. Cornish, R. L. Harries, D. Bosanquet, B. Rees, J. Ansell, N. Frewer, P. K. Dhruva Rao, C. Parry, R. Ellis-Owen, S. M. Phillips, C. Morris, J. Horwood, M. L. Davies, M. M. Davies, R. Hargest, Z. Davies, J. Hilton, D. Harris, A. Ben-Sassi, R. Rajagopal, D. Hanratty, S. Islam, A. Watkins, N. Bashir, S. Jones, I. R. Russell, J. Torkington, on behalf of the HART Trial Management Group

**Affiliations:** 1 Department of Surgery, University Hospital of Wales, Heath Park, Cardiff, CF14 4XW UK; 2 Princess of Wales Hospital, Bridgend, UK; 3 Morriston Hospital, Swansea, UK; 4 Wrexham Maelor Hospital, Wrexham, UK; 5 Glan Clwyd Hospital, Rhyl, UK; 6 Royal Glamorgan Hospital, Llantrisant, UK; 7 Swansea Clinical Trials Unit, Swansea University, Swansea, UK; 8 Involving People, Health and Care Research Wales, Cardiff, UK

**Keywords:** Incisional hernia, Abdominal closure, Hughes repair, Mass closure, Quality of life

## Abstract

**Background:**

Incisional hernias are common complications of midline closure following abdominal surgery and cause significant morbidity, impaired quality of life and increased health care costs.

The ‘Hughes Repair’ combines a standard mass closure with a series of horizontal and two vertical mattress sutures within a single suture. This theoretically distributes the load along the incision length as well as across it. There is evidence to suggest that this technique is as effective as mesh repair for the operative management of incisional hernias; however, no trials have compared the Hughes Repair with standard mass closure for the prevention of incisional hernia formation following a midline incision.

**Methods/design:**

This is a 1:1 randomised controlled trial comparing two suture techniques for the closure of the midline abdominal wound following surgery for colorectal cancer. Full ethical approval has been gained (Wales REC 3, MREC 12/WA/0374). Eight hundred patients will be randomised from approximately 20 general surgical units within the United Kingdom. Patients undergoing open or laparoscopic (more than a 5-cm midline incision) surgery for colorectal cancer, elective or emergency, are eligible. Patients under the age of 18 years, those having mesh inserted or undergoing musculofascial flap closure of the perineal defect in abdominoperineal wound closure, and those unable to give informed consent will be excluded. Patients will be randomised intraoperatively to either the Hughes Repair or standard mass closure. The primary outcome measure is the incidence of incisional hernias at 1 year as assessed by standardised clinical examination. The secondary outcomes include quality of life patient-reported outcome measures, cost-utility analysis, incidence of complete abdominal wound dehiscence and C-POSSUM scores. The incidence of incisional hernia at 1 year, assessed by computerised tomography, will form a tertiary outcome.

**Discussion:**

A feasibility phase has been completed. The results of the study will be used to inform current and future practice and potentially reduce the risk of incisional hernia formation following midline incisions.

**Trial registration:**

Trial Registration Number: ISRCTN 25616490. Registered on 1 January 2012.

**Electronic supplementary material:**

The online version of this article (doi:10.1186/s13063-016-1573-0) contains supplementary material, which is available to authorized users.

## Background

Incisional hernias (IHs) are ‘abdominal wall gaps around postoperative scars, perceptible or palpable by clinical examination or imaging’ [1, 2]. They are common complications of midline closure following major abdominal surgery and cause significant morbidity, impaired quality of life (QoL) [3] and increased cost [4]. The standard technique for abdominal closure is ‘mass closure’ (closing all layers of the abdominal wall, excluding the skin), usually with nonabsorbable sutures, although ‘slow-resorbing’ sutures such as polydioxanone (PDS) are also widely used [5].

The reported incidence of IHs ranges widely; from 8.6 to 33 % following open colorectal surgery, and from 4.7 to 24.3 % following laparoscopic colorectal surgery [6–9]. The long-term results of IH repair are disappointing. The two main surgical options for fixing these hernias are suture repair or mesh repair (suture closure reinforced by a synthetic mesh), yet recurrence rates are as high as 12 to 54 % and 2 to 36 %, respectively [10, 11]. IH repair may also lead to serious complications such as enterocutaneous fistulae, bowel obstruction or chronic pain, which have an even greater impact on QoL. Given such disappointing results from corrective surgery, the search for preventative measures is important.

Many factors contribute to the pathogenesis of IHs; these include diabetes mellitus [12], obesity [12, 13], cachexia [14], aged older than 45 years [13], male sex [13, 15], history of chronic obstructive pulmonary disease (COPD) [14, 16] post-menopausal status [17], history of abdominal aortic aneurysm [18], anaemia [14], history of smoking [15] and certain medications (e.g. corticosteroids) [19]. Most of these are outside the surgeons’ control and the only modifiable factors identified as having a substantial impact on IH rates are the surgical technique and the material used to close the abdominal wall musculofascial layer.

There have been many studies to identify the best technique for abdominal wall closure, yet there is still uncertainty about this. For example, the meta-analyses by Hodgson et al. [20], van’t Riet et al. [11] and Weiland et al. [21] concluded that nonabsorbable sutures reduce IH risk, whilst the more recent meta-analysis by Diener et al. [10] showed that absorbable sutures were associated with a lower risk. Such a discrepancy may be due in part to different inclusion or exclusion criteria. Furthermore, most studies included in these meta-analyses recruited small numbers of patients and lacked sufficient power to detect statistically significant differences between groups [10]. More recent work has focussed on different techniques used to close the abdominal wall. The STITCH trial [22], a Dutch multicentre, randomised controlled trial (RCT) that has reported its outcomes for 560 patients comparing small-stitch continuous sutures with (large-stitch) standard mass closure. They found a reduction from 21 % (large-bite) to 13 % (small-bite) in the rate of IHs at 1 year. The CONTinuous versus INTerrupted abdominal wall closure after emergency midline laparotomy (CONTINT) RCT, still recruiting, is comparing continuous with interrupted sutures in closing midline incisions after emergency laparotomy [23].

### Hughes Repair

**Fig. 1:**
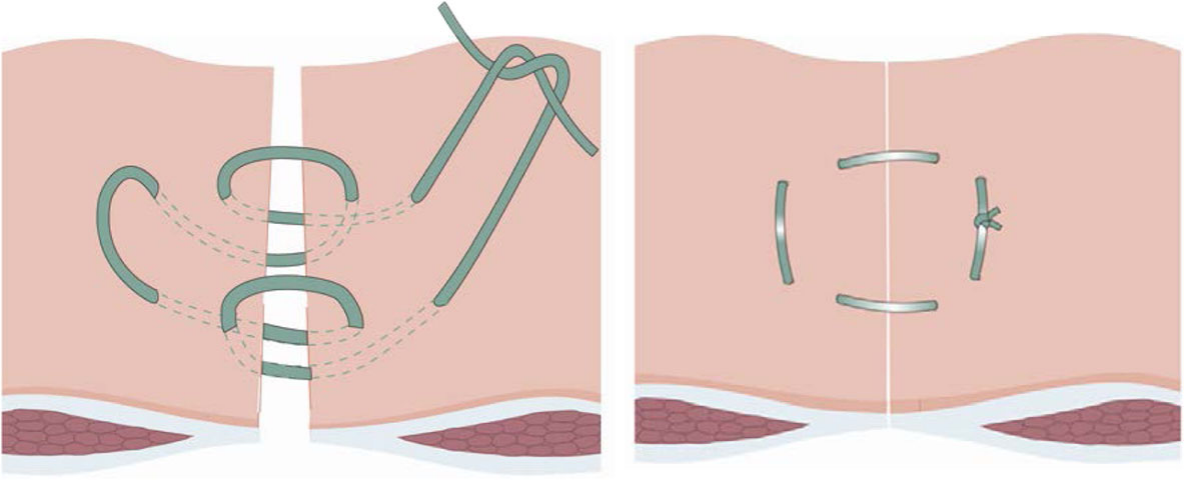
Diagram showing the Hughes closure method, using a combination of standard mass closure with a series of horizontal and two vertical mattress sutures within a single suture. When the sutures are pulled to close the defect, the sutures lie both across and along the incision

The eponymously titled ‘Hughes Repair’ (Professor Leslie Hughes, 1932–2011 [24]), also known as the ‘far-and-near’ or ‘Cardiff Repair’ [25] combines a standard mass closure (two loop 1-PDS sutures) with a series of horizontal and two vertical mattress sutures within a single suture (1 Nylon); theoretically distributing the load along the incision length as well as across it (Fig. 1). The principles are:

1. To ensure, by palpation, that only sound normal tissues are used for the repair
2. To use graduated tension for easy approximation
3. Use a monofilament Nylon suture, which has the advantage of slipping easily through tissues to create a pulley system [26]

The Hughes Repair has been demonstrated to be as effective as the standard mesh repair in treating patients with IHs [27]. It is also used for closing abdomens when patients are at high risk of IHs, after complete abdominal wound dehiscence and laparostomy [28]. This trial aims to ascertain if this technique can be used as primary prevention for IH formation. In addition it will provide valuable information on the aetiology of IH with an objective, radiological assessment of their formation.

## Methods/design

### Primary outcome

The primary outcome measure is to the incidence of IHs at 1 year from colorectal cancer surgery between the Hughes Repair and standard mass closure as assessed by clinical examination.

### Secondary outcomes

The secondary outcomes include:

- To compare QoL over 1 year between the Hughes Repair and standard mass closure (the Functional Assessment of Cancer Therapy-Colorectal (FACT-C) and the 12-Item Short-Form Health Survey (SF12))
- To evaluate the cost-effectiveness of the Hughes Repair relative to standard mass closure over the first year (using the Client Service Receipt Inventory)
- To test whether the Hughes Repair reduces the incidence of postoperative complete abdominal wound dehiscence between the Hughes Repair and standard mass closure by day 30
- To identify and characterise patient and surgical factors which increase the risk of developing IHs
- To estimate the prevalence of IHs at 1 year following surgery for colorectal cancer in patients receiving the Hughes Repair or standard mass closure

### Tertiary outcome

- To assess the incidence of IH at 1 year by computerised tomography (CT) scanning and compare to the clinical assessment

### Primary hypothesis

The Hughes Repair results in a reduced incidence of IH at 1 year in patients having midline abdominal wall closure incisions following elective or emergency colorectal cancer surgery when compared with standard mass closure.

### Study design

**Fig. 2:**
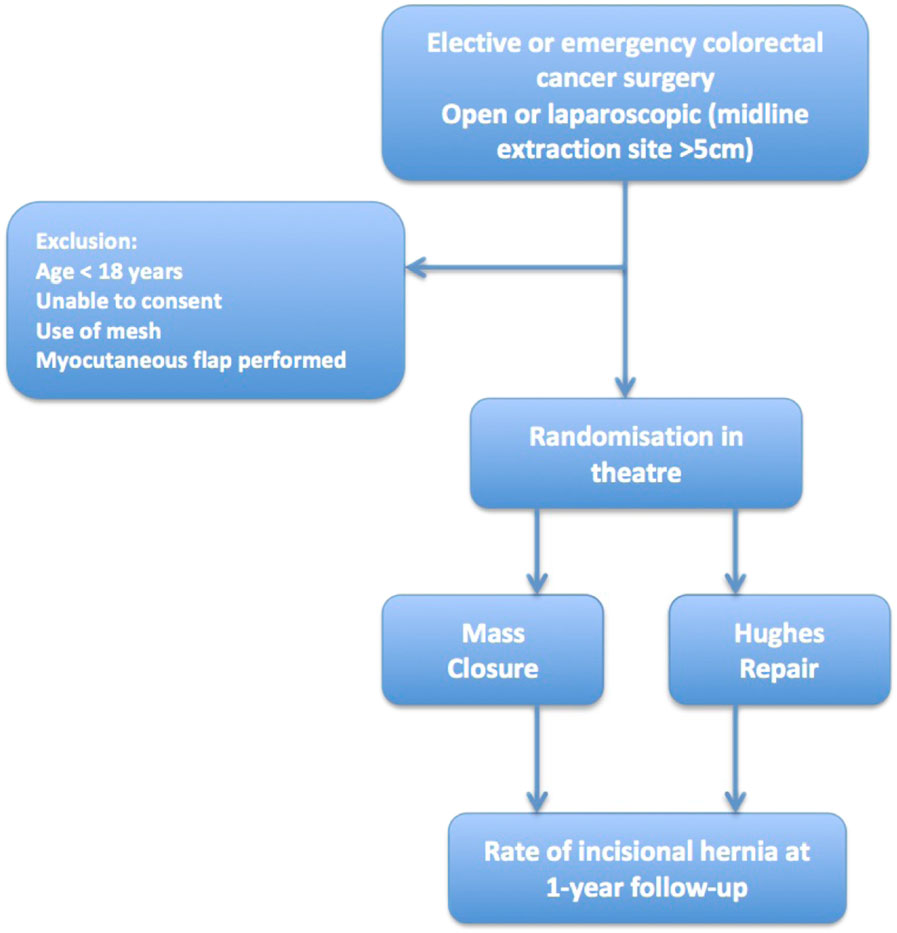
Hughes Abdominal Repair Trial (HART) study design

This is a multicentre, blinded, RCT (Fig. 2).

### Study population

The study will identify patients who are due to receive abdominal surgery for the treatment of colorectal cancer. Patients undergoing emergency surgical treatment as well as patients receiving elective surgical treatment will be eligible for inclusion.

### Setting

The study is being performed in general surgical units within the NHS across the United Kingdom, aiming for recruitment from at least 20 sites. Informed consent will be obtained from all patients. This study has ethical approval from Wales REC 3 (MREC 12/WA/0374). A full list of current approvals is appended.

### Eligibility criteria

#### Inclusion criteria

At screening:

- Patients aged 18 years or older
- Able to give informed consent
- Both standard mass closure and the Hughes Repair closure are suitable closing techniques for the patient
- An elective patient for colorectal cancer surgery following full staging investigations including an abdominal CT scan *or* an emergency patient with a strong suspicion of colorectal cancer as per CT

At point of surgical closure/randomisation:

- Midline abdominal incision (open or laparoscopic assisted/converted)
- Incision of 5 cm or more

#### Exclusion criteria

At screening:

- Unable to provide informed consent

At point of surgical closure/randomisation:

- Inserting a mesh as part of abdominal closure
- Undergoing musculofascial flap closure of perineal defect in abdominoperineal wound closure

### Outcome assessment

#### Primary outcome

The primary outcome is the incidence of IHs over 1 year as assessed by clinical examination of the abdomen. The clinical presence of a hernia will be assessed either by a surgeon or a nurse specialist who has received clinical examination training as part of their role. The presence of a hernia can be detected as a reducible, palpable mass, usually with a cough impulse, which may cause the patient discomfort or pain. The examiner will assess the patient ensuring to include the following:

- With the patient in a standing position, palpate the length of the closed wound and ask the patient to cough or perform the Valsalva manoeuvre
- With the patient in a supine position, palpate the length of the closed wound and ask the patient to cough or perform the Valsalva manoeuvre

#### Secondary outcome

The following secondary outcomes will be assessed:

- Two QoL Patient-reported Outcome Measures (PROMs) will be administered at baseline, 30 days, 6 months and 1 year to assess the differences between the two trial groups. The questionnaires used will be SF12 [29] and the FACT-C [30]
- Cost-utility analysis of the Hughes Repair in relation to the mass closure from the perspective of the NHS will be undertaken
- Data on the incidence of full-thickness abdominal wall dehiscence will be collected up to 30 days post operation, as well as details of any repair surgery and the closing sutures used
- Data will be collected regarding patient conditions that are considered to be associated with an increased risk of developing hernias. Colorectal – Physiological and Operative Severity Score for Understanding Mortality and Morbidity (C-POSSUM) scores [31] to assess risk of mortality and morbidity in patients undergoing colorectal surgery will also be completed. Data will be collected for patients developing SSIs (surgical site infections) in hospital; the SSIs will be classified into superficial, deep (involving muscle or fascia) or confined to an organ or space [32]
- The prevalence of IHs at 1 year as measured by clinical examination will be assessed. PROMs will be administered at baseline, 30 days, 6 months and 1 year
- The QoL of patients with or without IHs will be compared over 1 year. PROMs will be administered at baseline, 30 days, 6 months and 1 year to assess the differences between the two groups

### Sample size estimation

The study aims to detect a reduction in IH rates from 30 % for mass closure to 20 % for the Hughes Repair. To give 80 % power of detecting this difference with a 5 % significance level requires 640 patients to be followed up for 1 year. As loss to follow-up from similar trials [33] is about 20 % at 1 year, HART aims to recruit 800 patients in total.

## Discussion

### Study process

After screening, consent and surgery, each participant will attend two separate visits (at 30 days and 6 months) during the first year (these may be conducted by telephone if required) and undergo a CT scan and clinical examination at 1 year post surgery. Data collected at the 1-year visit will support the primary endpoint.

### Randomisation

An adaptive randomisation design will be used to allocate eligible patients to groups of similar size [34]. Telephone randomisation will be accessed by the closing surgeon and will take place during surgery and as close as possible to the time when the surgeon commences closure.

### Data management

This data management aspect of the study is being supported by the Swansea Trials Unit (STU). Data will be collected using an electronic data capture system (MACRO 4).

### Training in closure techniques

To assure the quality of the repair techniques, all surgeons participating in the trial (consultants and trainees) will complete training and quality assessment on the Hughes Repair. All participating surgeons will be assessed by the chief investigator or a designated assessor and approved only when closure technique is satisfactory. A reference instructional video will also be provided as well as ongoing quality review of the technique throughout the course of the trial. To monitor the training of professionals contributing to HART, a log will be maintained at each site with details of training, both surgical and in research governance notably Good Clinical Practice (GCP). For the purposes of the study mass closure will be taken to be the responsible consultant surgeon’s standard closure technique (Additional file 1).

### Radiological evaluation of incisional hernia

Dedicated trial radiologists will determine whether there is a hernia present, define it as herniation of the bowel or other intra-abdominal content outside the abdominal wall, and identify the presence of other hernias and the quality of the recti muscle.

### CT imaging

Scans should be acquired using the thinnest slice thickness capability of the scanner and images for review reconstructed to 5-mm or 2.5-mm slice thickness in the axial plane. Scans should be done using the standard departmental protocol for staging and follow-up scans.

### Transfer of CT images

The transfer of CT images from participating site to reviewing radiologists will be done using the Picture Archiving and Communications System (PACS) or equivalent. Relevant images will be requested of the study team at site on a regular basis.

### Management and safety

Documentation will be put in place to describe all key processes (governed by STU Standard Operating Procedures (SOPs)). The Trial Management Group (TMG), including patient and public representatives, will meet every month with audio facilities for site principal investigators. Protocol deviations and adverse events will be monitored by STU, regularly reported to the TMG with the clinical chief investigator taking overall responsibility, and formally reporting to the Data Monitoring Committee (DMC). Both the DMC and the Trial Steering Committee (TSC) will meet regularly to monitor progress.

### Data analysis

Data analysis will follow the principles outlined in the Statistical Analysis Plan (SAP), which covers both clinical and cost-effectiveness analyses. Specifically, analyses, by ‘treatment allocated’, will adjust for significant factors and covariates, and use an NHS perspective on costs, assessed via incremental cost-effectiveness ratios (ICERs). The DMC will be asked to review and comment on this integrated plan, and to approve it prior to any analysis of the data. Analysts, who should remain blinded until the TSC deem otherwise, will then undertake a single main analysis at the end of the trial when all 1-year visits have been completed.

## Trial status

### Ethical considerations

This study complies with the World Medical Association Declaration of Helsinki (2013) and the principles of GCP. CT scans at years 1 and 2 are required as part of standard care and, therefore, compliance with IRMER approval is in place. The study will respect the rights of participating patients and ensure confidentiality of patient information. Patients undergoing surgery for colorectal cancer have an excellent support system through the specialist cancer nurses and the clinical team, as well as several charities and voluntary organisations. Should participants have additional questions about the trial, advice will be available from both within the research team and outside of the research team in the form of websites such as the NHS website page: Clinical trials and medical research – Joining a trial, found on http://www.nhs.uk/Conditions/Clinical-trials/Pages/Takingpart.aspx.

### Current status

The trial is split into three phases, a feasibility, pilot and main study phase. The feasibility phase of 30 patients at the host site has been completed. Data from this part of the study will not be included in the final analysis. The pilot phase of the study of 80 patients is complete and with no safety issues identified following review by the DMC the main study is ongoing with further sites opening now.

## Additional file

Additional file 1: SPIRIT figure_HARTR1. (DOCX 16 kb)

## Abbreviations

CONTINT: CONTinuous versus INTerrupted abdominal wall closure after emergency midline laparotomy
COPD: Chronic obstructive pulmonary disease
C-POSSUM: Colorectal – Physiological and Operative Severity Score for Understanding Mortality and Morbidity
CT: Computed tomography
DMC: Data Monitoring Committee
FACT-C: Functional Analysis of Cancer Therapy – Colorectal
GCP: Good Clinical Practice
HART: Hughes Abdominal Repair Trial
ICER: Incremental cost-effectiveness ratios
IH: Incisional hernia
IRMER: Ionising radiation (Medical Exposure) regulations 2000
NHS: National health service
PACS: Picture Archiving and Communications System
PDS: polydioxanone
PI: Principal investigator
PROMs: Patient-reported Outcome Measures
QoL: Quality of life
REC: Research Ethics Committee
SAP: Statistical Analysis Plan
SCTU: Swansea Clinical Trials Unit
SF12 or SF36: Short Form 12 or 36 (quality of life questionnaires)
SSI: surgical site infection
STITCH: Suture Techniques to reduce the Incidence of The inCisional Hernia (RCT)
TSC: Trial Steering Committee
TMG: Trial Management Group
