# Supplementary material for: Hughes Abdominal Repair Trial (HART) – Abdominal wall closure techniques to reduce the incidence of incisional hernias: study protocol for a randomised controlled trial
Source: Trials. 2016 Sep 15;17:454. doi: 10.1186/s13063-016-1573-0 (PMC5025615; doi:10.1186/s13063-016-1573-0)
Supplement: Additional file 1: — SPIRIT figure_HARTR1. (DOCX 16 kb) [file 13063_2016_1573_MOESM1_ESM.docx]

|  | **In advance of surgery** | **Day 1** | **(Up to day 30)** | **Discharge** | **Day 30^1^** | **6 month^1^** | **Year 1^1^** |
| --- | --- | --- | --- | --- | --- | --- | --- |
|  |  | **+/- 0** |  |  | **+/- 5 days** | **+/- 30 days** | **+/- 2 months** |
| **Screening^2^** | x |  |  |  |  |  |  |
| **Patient consent** | x |  |  |  |  |  |  |
| **Eligibility^3^** | x | x |  |  |  |  |  |
| **Demography** | x |  |  |  |  |  |  |
| **Computed Tomography** | X^4^ |  |  |  |  |  | x |
| **Hernia Clinical Examination** | x |  |  |  |  |  | x |
| **Drug History** | x |  |  |  |  |  |  |
| **Medical History** | x |  |  |  |  |  |  |
| **Surgical History** | x |  |  |  |  |  |  |
| **FACT-C questionnaire** | x |  |  |  | x | x | x |
| **SF-12 questionnaire** | x |  |  |  | x | x | x |
| **Randomisation** |  | x |  |  |  |  |  |
| **Abdominal surgery** |  | x |  |  |  |  |  |
| **(Reoperation^5^)** |  |  | (x) |  |  |  |  |
| **Wound closure details** |  | x | (x) |  |  |  |  |
| **Surgical information** |  | x | (x) |  |  |  |  |
| **C-POSSUM** |  | x | (x) |  |  |  |  |
| **SSI** |  |  |  | x |  |  |  |
| **Patient SSI Diary** |  |  |  | x | x^7^ |  |  |
| **Cancer staging** |  |  |  |  | x |  |  |
| **Cancer status** |  |  |  |  |  |  | x |
| **Surgical activity** |  |  |  |  |  |  | x |
| **Client Service Receipt Inventory (CSRI) questionnaire** | x |  |  |  |  | x | x |
| **SAE reporting^6^** |  | x |  | x | x |  |  |
| **Death** |  |  |  | x | x | x | x |

^1^All visit timings and windows are in relation to date of surgery. ^2^Screening can occur at any point up to surgery. ^3^Eligibility is established at screening, and requires confirmation at point of randomisation. ^4^The diagnostic pre-operative abdominal CT can be taken at any point prior to surgery. If a CT scan does not take place between the time window for the one and two year CT, please select the CT closest to the one and two year timepoint. ^5^Reoperation occurs if the wound requires reopening within 30 days of index operation. ^6^Information on all SAEs from point of consent to 30 days post intervention will be collected. If the patient requires the abdominal wound to be re-opened during admission, then all SAEs will be collected until 30 days post re-operation. ^7^If the patient is still in hospital at day 30, the SSI diary does not need to be completed
